# Acute Pancreatitis—III

**Published:** 1912-08-03

**Authors:** 


### ACUTE PANCREATITIS—III.

### Its Diagnosis and Surgical Treatment.

(Continued from p. 433.)

The peritoneal fluid may gravitate toward the pelvis. In several cases in which this was marked, tube drainage of the pelvic cavity, with the semi-erect position of the patient, has been successfully adopted.

When there is a pancreatic abscess, or an omental bursitis and peripancreatitis, free drainage, whether by the anterior or the posterior route, is the prime essential. When pus is present it is of still more importance than under any other circumstances to use free drainage by tubes and not to rely on tampons alone.

Some question has arisen as to the treatment of an accompanying cholelithiasis found at the time of an operation for acute pancreatitis. Dr. Deaver holds that when the patient's condition warrants it the underlying and really primary biliary condition should be corrected, and so any probability of again having to perform a serious laparotomy avoided. Often, as Noetzel has pointed out, the gravity of the pancreatic condition and the weakness of the patient will prevent completion of the operation; and in several reported cases, such as that of Stieda, the leaving of biliary calculi has apparently been no hindrance to the complete recovery of the patient from the pancreatic condition. If there is any doubt in the operator's mind as to the advisability of dealing with the accompanying cholelithiasis he had better defer. Two operations and recovery are preferable to one procedure with a fatality.

The preparatory treatment for operation must evidently be very brief in most cases of acute pancreatitis. It does not offer great possibilities. One can do little to help the patient to stand the shock of surgical intervention beyond the use of the ordinary stimulants, etc. Speed and the avoidance of shock and chilling on the table play a far more important *rôle* in helping the patient to ultimate recovery.

*Post-operative treatment* is the same as in all other serious laparotomy cases, with but few added features. Drainage should be left in until it has very evidently fulfilled its functions—until there is nothing more to drain.

Recently Wohlgemuth* has, by experiment and also clinically, shown that by placing patients who have been operated upon for pancreatitis upon a typical antidiabetic diet it is possible to so alter the pancreatic secretion that it becomes less active and irritating. This fact seems to be abundantly confirmed by other observers. Not only this, but by the same use of diet the healing of both recent and old pancreatic fistulæ is accelerated. The results in this direction have been truly remarkable. This is, perhaps, the only post-operative treatment that differs in any way from that of the other suppurating intra-abdominal lesions and abdominal sinuses.

---

* *Berl. Klin. Woch.* 1907 and 1908.

